# Rate of Sodium Correction and Osmotic Demyelination Syndrome in Severe Hyponatremia: A Meta-Analysis

**DOI:** 10.2478/jccm-2024-0030

**Published:** 2024-07-31

**Authors:** Xin Ya See, Yu-Cheng Chang, Chun-Yu Peng, Shih-Syuan Wang, Kuan-Yu Chi, Cho-Hung Chiang, Cho-Han Chiang

**Affiliations:** Unity Hospital, Rochester Regional Health, Rochester, NY, USA; Danbury Hospital, Danbury, CT, USA; SUNY Downstate Medical Center, Brooklyn, NY, USA; Jacobi Medical Center, Albert Einstein College of Medicine, Bronx, NY, USA; National Taiwan University Hospital, Taipei, Taiwan; Mount Auburn Hospital, Harvard Medical School, Cambridge, MA, USA

**Keywords:** hyponatremia, rapid correction, osmotic demyelination syndrome

## Abstract

**Introduction:**

Current guidelines recommend limiting the rate of correction in patients with severe hyponatremia to avoid severe neurologic complications such as osmotic demyelination syndrome (ODS). However, published data have been conflicting. We aimed to evaluate the association between rapid sodium correction and ODS in patients with severe hyponatremia.

**Materials and methods:**

We searched PubMed, Embase, Scopus, Web of Science, and Cochrane Central Register of Controlled Trials from inception to November 2023. The primary outcome was ODS and the secondary outcomes were in-hospital mortality and length of hospital stay.

**Results:**

We identified 7 cohort studies involving 6,032 adult patients with severe hyponatremia. Twenty-nine patients developed ODS, resulting in an incidence rate of 0.48%. Seventeen patients (61%) had a rapid correction of serum sodium in the first or any 24-hour period of admission. Compared with a limited rate of sodium correction, a rapid rate of sodium correction was associated with an increased risk of ODS (RR, 3.91 [95% CI, 1.17 to 13.04]; I^2^ = 44.47%; p = 0.03). However, a rapid rate of sodium correction reduced the risk of in-hospital mortality by approximately 50% (RR, 0.51 [95% CI, 0.39 to 0.66]; I^2^ = 0.11%; p < 0.001) and the length of stay by 1.3 days (Mean difference, −1.32 [95% CI, −2.54 to −0.10]; I^2^ = 71.47%; p = 0.03).

**Conclusions:**

Rapid correction of serum sodium may increase the risk of ODS among patients hospitalized with severe hyponatremia. However, ODS may occur in patients regardless of the rate of serum sodium correction.

## Introduction

Hyponatremia is one of the most common electrolyte disorders in hospitalized patients. Current guidelines, based on data reported by small retrospective case series, recommend limiting the rate of correction in patients with severe hyponatremia to avoid severe neurologic complications, most notably osmotic demyelination syndrome (ODS) [1, 2]. However, in a recent large cohort study, rapid correction of sodium was not associated with an increased risk of ODS in patients with severe hyponatremia [3]. Given this conflicting data, we conducted a systematic review and meta-analysis to evaluate the association between rapid sodium correction and ODS in patients with severe hyponatremia.

## Methods

We performed a comprehensive search in PubMed, Embase, Scopus, Web of Science, and Cochrane Central Register of Controlled Trials from inception to November 2023. The literature search was conducted using the patient, intervention, comparison, outcome (PICO) framework as summarized in **Supplemental Table 1**. We performed this study in accordance to the Preferred Reporting Items for Systematic Reviews and Meta-Analyses (PRISMA) guidelines [4]. We included randomized controlled trials or observational studies that reported adult patients admitted with severe hyponatremia, defined as serum sodium of less than 120 mmol/L on admission. We excluded case series or case reports. The primary outcome was ODS diagnosed by brainstem auditory evoked response [5], neuroimaging (magnetic resonance imaging) [3, 6,7,8,9], or pathology (postmortem examination) [5], and the secondary outcomes were in-hospital mortality and length of hospital stay. Rapid correction was defined as a sodium correction rate of more than 10 or 12 mmol/L within the first or any 24-hour period after admission [1, 2]. Two reviewers (XY See and YC Chang) independently extracted the study demographics and outcome data. Risk of bias was performed by two reviewers independently (XY See and CY Peng) using the Risk of Bias In Non-randomized Studies-of Interventions (ROBINS-I) tool [10]. In cases of disagreements, consensus was reached by discussing with the third reviewer (CHa Chiang). Meta-analysis was performed using a random-effects model based on the Hartung-Knapp-Sidik-Jonkman approach. Results were presented as forest plots with risk ratios (RRs) for dichotomous outcomes and mean differences for continuous outcomes. In studies reporting only the median, range, and size of the study, we approximated the means and standard deviations [11]. We also summarized the prevalence of patients who had one of the following risk factors associated with ODS: alcohol use disorder, hypokalemia, cirrhosis, or an initial serum sodium of less than 105 mmol/L on admission [1, 2]. All analyses were performed using STATA 16.0 (StataCorp, College Station, Texas).

## Results

**Fig. 1.**
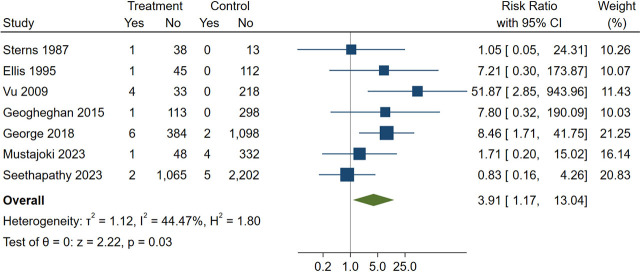
Forest plot showing the association between rapid serum sodium correction and osmotic demyelination syndrome

Our systematic review identified 7 cohort studies involving 6,032 adult patients with severe hyponatremia [3, 5,6,7,8,9, 12] (**Table 1 and Supplemental Figure 1**). No randomized controlled trials were identified. All studies had moderate or serious risk of bias (**Supplemental Table 2**). Five studies used 10 mmol/L/24 h [3, 6,7,8, 12] while one study used 12 mmol/L/24 h [9] as cutoffs for rapid correction of serum sodium. One study used 0.55 mmol/L/h to define rapid correction [5]. Among these studies, 29 patients developed ODS, resulting in an incidence rate of 0.48%. Only 28 out of 29 patients with osmotic demyelination syndrome had available data. Among 28 cases of ODS with available data, 14 (50%) were male, 21 (75%) had alcohol use disorder, 18 (64%) had hypokalemia on admission, 3 (11%) had liver cirrhosis, 9 (32%) had a serum sodium level of less than 105 mmol/L on admission, and 17 (61%) had a rapid correction of serum sodium in the first or any 24-hour period (**Supplemental Table 3**). Twenty-five cases of ODS (89%) had at least one of the following risk factors: alcohol use disorder, hypokalemia on admission, cirrhosis, or serum sodium level of less than 105 mmol/L on admission. The remaining 3 ODS cases (Patient 3, 26 and 27) without these risk factors did not experience a rapid correction of serum sodium. Compared with a limited rate of sodium correction, a rapid rate of sodium correction was associated with an increased risk of ODS (RR, 3.91 [95% CI, 1.17 to 13.04]; I^2^ = 44.47%; p = 0.03) (**Figure 1**). However, a rapid rate of sodium correction reduced the risk of in-hospital mortality by approximately 50% (RR, 0.51 [95% CI, 0.39 to 0.66]; I^2^ = 0.11%; p < 0.001) and the length of stay by 1.3 days (Mean difference, −1.32 [95% CI, −2.54 to −0.10]; I^2^ = 71.47%; p = 0.03) compared to a limited rate of sodium correction (**Supplemental Figure 2 and 3**).

**Table 1.** Study characteristics

**Trial (Study, Year)**	**Number of patients**	**Country**	**Study design**	**Inclusion criteria**	**Rapid sodium correction definition**	**Cases of ODS, n (%)**	**Diagnosis of ODS**
Sterns, 1987	62	USA	Retrospective cohort	Patients with serum sodium concentration of ≤110 mmol/L	The rate of correction to increase the serum sodium to ≥120 mmol/L exceeds 0.55 mmol/L/h	3 (4.8)	Postmortem examination, brainstem auditory evoked responses
Ellis, 1995	184	USA, UK	Retrospective cohort	All adult patients (≥16 years) with serum sodium ≤120 mmol/L at any stage of their hospitalization	>10 mmol/L/24h	1 (0.5)^a^	MRI
Vu, 2009	247	Australia	Retrospective cohort	All patients with serum sodium ≤120 mmol/L on admission	>12 mmol/L within the first 24h	4 (1.6)	MRI
Geogheghan, 2015	412	USA	Retrospective cohort	All hospitalized patients with serum sodium ≤120 mmol/L after correction for serum glucose	>10 mmol/L/24h	1 (0.2)	Clinically or radiographically confirmed^b^
George, 2018	1490	USA	Retrospective cohort	Adults ≥18 years of age admitted with an initial serum sodium <120mEq/L	>8 mEq/L/24h, >10 mEq/L/24h, or >18 mEq/L/48h^c^	9 (0.6)^d^	MRI
Mustajoki, 2023	363	Finland	Retrospective cohort	All patients admitted to the ED with plasma sodium concentrations <116 mmol/L	>10 mmol/L/24h	5 (1.4)	MRI
Seethapathy, 2023	3274	USA	Retrospective cohort	Patients (at least 18 years of age) hospitalized with serum sodium level <120mEq/L in the 24 hours preceding or 24 hours after the recorded time of admission	>10 mEq/L/24h	7 (0.2)	MRI

a There is no detailed information available about the patient with ODS;

b No detailed information is available regarding the clinical or radiographic diagnosis of ODS. There was only one patient diagnosed with ODS in this study;

c The study has different data available for different definitions of rapid correction;

d One patient had ODS occurring before hospitalization with severe hyponatremia and was excluded from our table 1.

## Discussion

In this systematic review and meta-analysis, ODS occurred in less than 5 per 1000 patients with an initial serum sodium of less than 120 mmol/L. A sodium correction rate of more than 10 or 12 mmol/L within the first or any 24-hour period after admission appeared to increase the risk of ODS by nearly 4-fold though it also led to a reduction in in-hospital mortality and length of stay among patients hospitalized with severe hyponatremia. Importantly, ODS was observed in both rapid and limited correction of serum sodium. Furthermore, the majority (89%) of the ODS cases occurred in patients with a history of alcohol use disorder, associated hypokalemia, cirrhosis, or an initial serum sodium of less than 105 mmol/L, regardless of the rate of serum sodium correction.

This study has several limitations. All the included studies were observational, and as such there were likely baseline differences between the groups who underwent rapid and limited sodium correction. Because we did not have access to patient-level data, we were unable to adjust for potential underlying confounders. These differences could either underestimate or overestimate the association between the rate of sodium correction and ODS. Furthermore, it was unclear if the included patients had chronic or acute hyponatremia. Current guidelines on limiting the rate of serum sodium correction apply to patients with chronic hyponatremia (defined as hyponatremia for more than 48 hours) in which the risk of ODS in rapid correction is postulated to be greater [1, 2]. Finally, these results only apply to patients with an initial serum sodium of less than 120 mmol/L.

In conclusion, rapid correction of serum sodium may increase the risk of ODS among patients hospitalized with severe hyponatremia. Nevertheless, ODS typically occurs in patients with predisposing risk factors regardless of the rate of serum sodium correction. Existing evidence is based on observational studies which are susceptible to biases and well-designed randomized controlled trials are needed to validate these results.
